# Early renoprotection by anemia correction

**DOI:** 10.1007/s10157-018-1554-6

**Published:** 2018-03-10

**Authors:** Satoru Kuriyama

**Affiliations:** Jikei University School of Medicine, 3-25-8, Nishi-shinbashi, Minato-ku, Tokyo, 105-8461 Japan

*To the Editor*

Whether correction of anemia by erythropoiesis-stimulating agent (ESA) retards the progression of chronic kidney disease (CKD) is still a matter for debate. The early study suggested that correction of anemia with ESA was beneficial to slow down the CKD progression [1]. In contrast, the studies CREATE, CHOIR, which compared high target group vs. low target group, did not support such a renoprotection [2, 3]. Of interest is that the recent study suggests that anemia treatment with ESA has a substantial renal preserving effect over time [4].

Taking all of these into consideration, Covic et al. conducted a meta-analysis on 19 studies using different endpoints and insisted that ESA does not prevent the progression of CKD [5]. I agree in general that CKD patients do not always benefit from treatment with ESA on renoprotection. One must be careful, however, about renoprotection at an early stage because renal anemia develops at an advanced phase in most of the CKD patients.

In a daily clinical practice of examining patients with early phase CKD, we nephrologists are instinctively aware of the fact that there are subgroups of patients who respond well to the therapy with ESA, and as a consequence slow the progression. The characteristics of those patients may include CKD at an early stage, non-diabetic, younger age, and those with less proteinuria. Covic’s review was scientific enough to discuss the flaw that there was an absence of poor early outcomes such as doubling of serum creatinine (Cr) concentration, an early surrogate marker for the progression. In fact, high hemoglobin apparently favors doubling of Cr in their analysis [5]. In this context, the research on the early phase prevention of failing kidney by ESA is probably in dire need.

## References

[1] Kuriyama S, Tomonari H, Yoshida H, Hashimoto T, Kawaguchi Y, Sakai O (1997). Reversal of anemia by erythropoietin therapy retards the progression of chronic renal failure, especially in nondiabetic patients. Nephron.

[2] Drüeke TB, Locatelli F, Clyne N, Eckardt KU, Macdougall IC, Tsakiris D, Burger HU, Scherhag A, CREATE Investigators (2006). Normalization of hemoglobin level in patients with chronic kidney disease and anemia. N Engl J Med.

[3] Singh AK, Szczech L, Tang KL, Barnhart H, Sapp S, Wolfson M, Reddan D, CHOIR Investigators (2006). Correction of anemia with epoetin alfa in chronic kidney disease. N Engl J Med.

[4] Tsubakihara Y, Gejyo F, Nishi S, Iino Y, Watanabe Y, Suzuki M, Saito A, Akiba T, Hirakata H, Akizawa T (2012). High target hemoglobin with erythropoiesis-stimulating agents has advantages in the renal function of non-dialysis chronic kidney disease patients. Ther Apher Dial.

[5] Covic A, Nistor I, Douciu MD, Dumea R, Bolignanob D, Goldsmith D (2014). Erythropoiesis-stimulating agents (ESA) for preventing the progression of chronic disease: a meta-analysis of 19 studies. Am J Nephrol.

